# Pathological Complete Response in Patients With Resected Pancreatic Adenocarcinoma After Preoperative Chemotherapy

**DOI:** 10.1001/jamanetworkopen.2024.17625

**Published:** 2024-06-18

**Authors:** Thomas F. Stoop, Atsushi Oba, Y. H. Andrew Wu, Laurel E. Beaty, Kathryn L. Colborn, Boris V. Janssen, Mohammed H. Al-Musawi, Salvador Rodriguez Franco, Toshitaka Sugawara, Oskar Franklin, Ajay Jain, Akio Saiura, Alain Sauvanet, Alessandro Coppola, Ammar A. Javed, Bas Groot Koerkamp, Braden N. Miller, Claudia E. Mack, Daisuke Hashimoto, Damiano Caputo, Dyre Kleive, Elisabetta Sereni, Giulio Belfiori, Hirofumi Ichida, Jacob L. van Dam, Jeanne Dembinski, Keiichi Akahoshi, Keith J. Roberts, Kimitaka Tanaka, Knut J. Labori, Massimo Falconi, Michael G. House, Motokazu Sugimoto, Minoru Tanabe, Naoto Gotohda, Paul S. Krohn, Richard A. Burkhart, Rohan G. Thakkar, Rupaly Pande, Safi Dokmak, Satoshi Hirano, Stefan K. Burgdorf, Stefano Crippa, Stijn van Roessel, Sohei Satoi, Steven A. White, Thilo Hackert, Trang K. Nguyen, Tomohisa Yamamoto, Toru Nakamura, Vismaya Bachu, William R. Burns, Yosuke Inoue, Yu Takahashi, Yuta Ushida, Zohra V. Aslami, Caroline S. Verbeke, Arantza Fariña, Jin He, Johanna W. Wilmink, Wells Messersmith, Joanne Verheij, Jeffrey Kaplan, Richard D. Schulick, Marc G. Besselink, Marco Del Chiaro

**Affiliations:** 1 Division of Surgical Oncology, Department of Surgery, University of Colorado, Anschutz Medical Campus, Aurora; 2 Amsterdam UMC, University of Amsterdam, Department of Surgery, Amsterdam, the Netherlands; 3 Cancer Center Amsterdam, Amsterdam, the Netherlands; 4 Department of Hepatobiliary and Pancreatic Surgery, Cancer Institute Hospital, Japanese Foundation for Cancer Research, Ariake, Tokyo, Japan; 5 Department of Hepatobiliary and Pancreatic Surgery, Graduate School of Medicine, Tokyo Medical and Dental University, Tokyo, Japan; 6 Division of Hepatobiliary and Pancreatic Surgery, Johns Hopkins University School of Medicine, Baltimore, Maryland; 7 The Sidney Kimmel Comprehensive Cancer Center at Johns Hopkins University, Baltimore, Maryland; 8 Department of Biostatistics and Informatics, Colorado School of Public Health, University of Colorado Anschutz Medical Campus, Aurora; 9 Department of Medicine, University of Colorado Anschutz Medical Campus, Aurora; 10 Adult and Child Center for Outcomes Research and Delivery Science, University of Colorado Anschutz Medical Campus, Aurora; 11 Amsterdam UMC, University of Amsterdam, Department of Pathology, Amsterdam, the Netherlands; 12 Clinical Trials of Office, Department of Surgery, University of Colorado, Anschutz Medical Campus, Aurora; 13 Department of Diagnostics and Intervention, Surgery, Umeå University, Umeå, Sweden; 14 Division of Surgical Oncology, Stephenson Cancer Center, University of Oklahoma Health Sciences Center, Oklahoma City; 15 Department of Hepatobiliary-Pancreatic Surgery, Juntendo University School of Medicine, Tokyo, Japan; 16 Department of Surgery, Hôpital Beaujon, Clichy, France; 17 Dipartimento di Chirurgia Sapienza Università di Roma, Rome, Italy; 18 Division of Surgical Oncology, Department of Surgery, New York University Medical Center, New York, New York; 19 Department of Surgery, Erasmus MC Cancer Institute, Rotterdam, the Netherlands; 20 Department of General, Visceral and Transplantation Surgery, Heidelberg University Hospital, Heidelberg, Germany; 21 Department of Surgery, Kansai Medical University, Osaka, Japan; 22 Fondazione Policlinico Universitario Campus Bio-Medico, Rome, Italy; 23 Research Unit of General Surgery, Department of Medicine and Surgery, Università Campus Bio-Medico di Roma, Rome, Italy; 24 Department of Hepato-Pancreato-Biliary Surgery, Oslo University Hospital and Institute of Clinical Medicine, University of Oslo, Oslo, Norway; 25 Unit of General and Pancreatic Surgery, The Pancreas Institute, University of Verona Hospital Truty, Verona, Italy; 26 Pancreatic and Transplant Surgery Unit, San Raffaele Hospital IRCCS, Vita-Salute University, Milano, Italy; 27 Hepato-Pancreato-Biliary Unit, Department of Surgery, University Hospitals of Birmingham, Birmingham, UK; 28 Department of Gastroenterological Surgery II, Hokkaido University, Faculty of Medicine, Hokkaido, Japan; 29 Department of Surgery, Indiana University School of Medicine, Indianapolis; 30 Department of Hepatobiliary and Pancreatic Surgery, National Cancer Center Hospital East, Kashiwa, Japan; 31 Department of Surgery and Transplantation, Copenhagen University Hospital, Copenhagen, Denmark; 32 Department of Hepato-Pancreatico-Biliary and Transplant Surgery, Freeman Hospital, Newcastle University, Newcastle upon Tyne, UK; 33 Department of General, Visceral and Thoracic Surgery, University Hospital Hamburg-Eppendorf, Hamburg, Germany; 34 Department of Pathology, Oslo University Hospital, University of Oslo, Oslo, Norway; 35 Amsterdam UMC, University of Amsterdam, Department of Medical Oncology, Amsterdam, the Netherlands; 36 Division of Medical Oncology, Department of Medicine, University of Colorado School of Medicine, Aurora; 37 Department of Pathology, University of Colorado School of Medicine, Aurora

## Abstract

**Importance:**

Preoperative chemo(radio)therapy is increasingly used in patients with localized pancreatic adenocarcinoma, leading to pathological complete response (pCR) in a small subset of patients. However, multicenter studies with in-depth data about pCR are lacking.

**Objective:**

To investigate the incidence, outcome, and risk factors of pCR after preoperative chemo(radio)therapy.

**Design, Setting, and Participants:**

This observational, international, multicenter cohort study assessed all consecutive patients with pathology-proven localized pancreatic adenocarcinoma who underwent resection after 2 or more cycles of chemotherapy (with or without radiotherapy) in 19 centers from 8 countries (January 1, 2010, to December 31, 2018). Data collection was performed from February 1, 2020, to April 30, 2022, and analyses from January 1, 2022, to December 31, 2023. Median follow-up was 19 months.

**Exposures:**

Preoperative chemotherapy (with or without radiotherapy) followed by resection.

**Main Outcomes and Measures:**

The incidence of pCR (defined as absence of vital tumor cells in the sampled pancreas specimen after resection), its association with OS from surgery, and factors associated with pCR. Factors associated with overall survival (OS) and pCR were investigated with Cox proportional hazards and logistic regression models, respectively.

**Results:**

Overall, 1758 patients (mean [SD] age, 64 [9] years; 879 [50.0%] male) were studied. The rate of pCR was 4.8% (n = 85), and pCR was associated with OS (hazard ratio, 0.46; 95% CI, 0.26-0.83). The 1-, 3-, and 5-year OS rates were 95%, 82%, and 63% in patients with pCR vs 80%, 46%, and 30% in patients without pCR, respectively (*P* < .001). Factors associated with pCR included preoperative multiagent chemotherapy other than (m)FOLFIRINOX ([modified] leucovorin calcium [folinic acid], fluorouracil, irinotecan hydrochloride, and oxaliplatin) (odds ratio [OR], 0.48; 95% CI, 0.26-0.87), preoperative conventional radiotherapy (OR, 2.03; 95% CI, 1.00-4.10), preoperative stereotactic body radiotherapy (OR, 8.91; 95% CI, 4.17-19.05), radiologic response (OR, 13.00; 95% CI, 7.02-24.08), and normal(ized) serum carbohydrate antigen 19-9 after preoperative therapy (OR, 3.76; 95% CI, 1.79-7.89).

**Conclusions and Relevance:**

This international, retrospective cohort study found that pCR occurred in 4.8% of patients with resected localized pancreatic adenocarcinoma after preoperative chemo(radio)therapy. Although pCR does not reflect cure, it is associated with improved OS, with a doubled 5-year OS of 63% compared with 30% in patients without pCR. Factors associated with pCR related to preoperative chemo(radio)therapy regimens and anatomical and biological disease response features may have implications for treatment strategies that require validation in prospective studies because they may not universally apply to all patients with pancreatic adenocarcinoma.

## Key Points

**Question:**

What are the incidence, outcome, and associated factors of pathological complete response (pCR) in patients with resected pancreatic adenocarcinoma after chemo(radio)therapy?

**Findings:**

This cohort study of 1758 patients found a pCR rate of 4.8%, which was associated with longer overall survival compared with no pCR. Factors associated with pCR included preoperative (modified) FOLFIRINOX, preoperative radiotherapy (particularly stereotactic body radiation therapy), radiologic response, and normal(ized) serum carbohydrate antigen 19-9.

**Meaning:**

Although pCR does not reflect cure, these findings suggest that it is associated with improved OS, and the identified factors associated with pCR may have implications for treatment strategies.

## Teaser

This cohort study analyzes the incidence, outcome, and factors associated with pathological complete response in patients with pancreatic adenocarcinoma who underwent preoperative chemo(radio)therapy followed by surgical resection.

## Introduction

The treatment of localized pancreatic adenocarcinoma has evolved during the past decade with the increasing use of preoperative chemo(radio)therapy, particularly multiagent chemotherapeutic regimens.^1^ Preoperative therapy provides the chance for improved disease control while selecting patients with more favorable tumor biology for surgical resection,^2^ leading to resection rates of 77%, 61%, and 22% among patients with primary resectable, borderline resectable, and locally advanced pancreatic cancer, respectively.^3^

Nevertheless, the interpretation of anatomical, biological, and conditional parameters for personalized restaging remains challenging,^4,5,6^ illustrated by high early recurrence rates.^7,8^ Histopathological residual tumor burden after resection following preoperative therapy is 1 of the biological parameters for disease response.^9,10,11^ The presence and extent of vital tumor burden are considered a surrogate marker for the tumor’s response on preoperative therapy, which could be used for prognostication and may guide the decision-making for adjuvant therapy.^12^ Pathological complete response (pCR) is the ultimate tumor response, with an estimated incidence of 4%.^13^

Radiation therapy and longer duration of preoperative chemotherapy have been suggested to be associated with pCR.^14^ Pathological complete response is associated with improved overall survival (OS), with a median up to 100 months,^14,15,16,17^ although up to half of patients with pCR develop disease recurrence.^16,17,18^ However, evidence regarding pCR is based on large national databases with limited granularity or small single-center series.^13,14,15,16,19,20,21,22,23,24^ Better insight into the outcomes and factors associated with pCR may contribute to the improvement of preoperative therapy in patients with pancreatic adenocarcinoma, improving selection for surgery and prognostication. Therefore, the current international, observational, multicenter cohort study aimed to perform an in-depth analysis on the incidence, outcome, and factors associated with pCR within a large cohort of consecutive patients with pancreatic adenocarcinoma who underwent preoperative chemo(radio)therapy followed by surgical resection.

## Methods

This retrospective, observational, multicenter cohort study was performed in accordance with the Strengthening the Reporting of Observational Studies in Epidemiology (STROBE) guidelines.^25^ The study procedures were reviewed and approved by the Colorado Multiple Institutional Review Board at the University of Colorado. The need for informed consent was waived by the institutional review board because of the retrospective nature of this study.

### Study Design and Patients

All consecutive adult patients (aged ≥18 years) were retrospectively included from institutional databases in 19 centers from 8 countries who underwent any type of pancreatic resection after preoperative chemo(radio)therapy (January 1, 2010, to December 31, 2018) for localized pancreatic adenocarcinoma. Exclusion criteria included fewer than 2 cycles of preoperative chemotherapy, unknown type of preoperative chemotherapy, and pCR cases without preoperative cytologic or histologic test results classified as “suspicious for malignancy” or “positive/malignant.”^26^ The arbitrary cutoff of fewer than 2 cycles of preoperative chemotherapy was selected to approach the daily clinical practice by reducing the selection. Because of the differently used tumor regression grading classification,^27^ the interobserver variability,^28,29^ and differences in sampling strategies^12,30^ among centers and pathologists around the world, the current study focused on comparing patients with pCR vs without pCR. Data on patient race and ethnicity were not collected because it was not considered during the design of this study.

### Definitions

The American Society of Anesthesiologists Physical Status was used to indicate patients’ conditional status. Pancreatic adenocarcinoma was staged using the *TNM Classification of Malignant Tumours* (7th edition) and the National Comprehensive Cancer Network guideline, version 1.2019.^31,32^ If the tumor involved multiple anatomical locations in the pancreas (ie, head, body, and/or tail), the most proximal location was registered.

When a patient underwent a chemotherapy switch preoperatively, the dominant chemotherapeutic regimen was used, whereby other chemotherapy lines were registered as second-line chemotherapy. The following strategy was used to determine the dominant regimen. First, if both regimens were single-agent or multiagent chemotherapy but the number of administered cycles from both regimens was not available, the last regimen before surgery was considered as the dominant regimen. If the number of cycles from all regimens was available, the chemotherapeutic regimen with the most administered cycles was used as the dominant regimen. Second, if multiple lines were given, including a multiagent and single-agent chemotherapy, the multiagent chemotherapy was defined as the dominant regimen, regardless of the order or number of cycles. Third, systemic chemotherapy was considered superior to intraperitoneal-administered chemotherapy. Additional experimental drugs were not taken into account. The interval between the start of preoperative chemotherapy and surgery was used as a surrogate marker for the preoperative treatment duration. The duration was stratified into less than 4, 4 to less than 6, 6 or more to less than 12, and 12 or more months.

Radiological response evaluation was defined in accordance with the Response Evaluation Criteria in Solid Tumors (RECIST) criteria.^33^ A carbohydrate antigen 19-9 (CA 19-9) level of 37 U/mL or greater and a carcinoembryonic antigen (CEA) level greater than 5 ng/mL (to convert to micrograms per liter, multiply by 1) were considered elevated.

Type and extent of pancreatic surgery were defined using the International Study Group for Pancreatic Surgery definition.^34^ Major morbidity was defined as a Clavien-Dindo grade of IIIa or higher within 90 days after surgery.^35^ Radicality (R0 vs R1) was classified following the Royal College of Pathologist definition.^36^ Pathological complete response was defined as the absence of any vital tumor tissue in the sampled pancreatic resection specimen.

Recurrence-free survival (RFS) and OS were measured from the time of surgery. Additionally, the OS measured from the start of preoperative chemotherapy was provided. From the date of surgery, follow-up was measured until death or the most recent date alive. Data collection ranged from February 1, 2020, to April 30, 2022, and analyses were performed between January 1, 2022, and December 31, 2023.

### Statistical Analysis

Data analyses were performed using R software, version 4.2.2 (R Foundation for Statistical Computing).^37^ Statistical significance was determined using a 2-sided *P* < .05. Patients with and without pCR were compared using descriptive statistics. Bivariable statistics were estimated using χ^2^ or Fisher exact (when observed cell counts were <5) tests for categorical data. Normally distributed continuous variables were compared using the Welch independent 2-sample *t* test, and the Wilcoxon rank sum test was used for nonnormally distributed data.

The amount of missing data per variable ranged from 0% to more than 50%. Data appeared to be missing not at random for larger counts of missing data. Therefore, a missing data category was introduced for each categorical variable that was missing 2% or more; otherwise, patients with missing data for variables missing less than 2% were simply quantified and displayed as counts in bivariable comparisons. If a categorical variable had less than 2% missing data, the missing patients were not included in the overall proportions reported, and they were not included in the test of association because of a lack of power. If a categorical variable had missing data of 2% or more, these patients were classified as missing and included in the overall proportions and the test of association. For all continuous variables, the missing data were not included in the test of association.

Serum CA 19-9 was treated as a categorical variable because there were substantial missing data for this variable (so we needed to include a missing data category) and because the association between this variable and the outcomes was nonlinear. Furthermore, the groupings chosen for this variable were considered clinically meaningful. For the relative change in serum CA 19-9 between diagnosis and restaging, the area under the curve method was used to select the optimal threshold among patients with elevated serum CA 19-9 at diagnosis who showed any degree of reduction.

The median RFS and OS times with 95% CIs were calculated using the Kaplan-Meier method, and subgroups were compared using the log-rank test using the survival package in R.^38,39^ Univariable (unadjusted) and multivariable (adjusted) Cox proportional hazards regression models were used to assess the association between clinical parameters and OS measured from surgery using the survival package in R.^38,39^ The OS from surgery was used so that the findings can be used for prognostication immediately after surgery, because pCR is only known at that time. The results are presented in hazard ratios (HRs) with 95% CIs.

Univariable and multivariable logistic regression models were used to assess factors associated with pCR using the stats package in R.^37^ The results are presented as odds ratios (OR) with 95% CIs. For both the Cox proportional hazards and logistic regression models, the following strategy was used. The univariable models included all variables that were considered clinically relevant based on the literature and clinical experience. Independent variables with *P* < .25 were included in the multivariable models.

Due to collinearity among the 3 serum CA 19-9 variables, only the serum CA 19-9 parameter with the strongest bivariable statistical association was tested in the multivariable analysis, based on *P* value. Again, missing data were included as a category if they were missing for at least 2% of patients. Otherwise, missing data were excluded for variables missing less than 2% of the time. In the bivariable analyses, if the only significant comparison within a categorical variable was for the missing group, the variable was not included in the multivariable analysis.

## Results

Overall, 1758 patients (mean [SD] age, 64 [9] years; 879 [50.0%] male and 879 [50.0%] female) underwent resection of pancreatic adenocarcinoma after chemo(radio)therapy and were included from 4 centers in the US (798 patients [45.4%]), 6 centers in Japan (366 patients [20.8%]), and 9 centers in Europe (594 patients [33.8%]). The number of patients per center ranged from 5 to 397. Of the 1758 included patients, 85 (4.8%) were diagnosed with pCR, with a median incidence of 3.8% (IQR, 0.3%-7.8%) per center. See eAppendix 1 in Supplement 1 for the incidences of pCR per center.

### Clinicopathological Details

At time of diagnosis, pancreatic adenocarcinoma was staged as primary resectable (n = 429 [24.5%]), borderline resectable (n = 856 [48.9%]), or locally advanced (n = 465 [26.6%]). The primary tumor was mostly located in the pancreatic head (n = 1276 [72.6%]). The median (IQR) serum CA 19-9 level before preoperative therapy was 183 (46-626) U/mL, without a difference between patients with or without pCR. See Table 1 for baseline characteristics at time of diagnosis.

**Table 1.** Baseline Characteristics at Diagnosis^a^

Characteristic	Overall cohort (N = 1758)	pCR (n = 85)	No pCR (n = 1673)	*P* value^b^
Age, mean (SD), y	64 (9)	62 (9)	64 (9)	.01^c^
Sex				
Female	879 (50.0)	41 (48.2)	838 (50.1)	.74^d^
Male	879 (50.0)	44 (51.8)	835 (49.9)	
ASA-PS				
I-II	1295 (74.0)	45 (52.9)	1250 (75.0)	<.001^d^
III-IV	456 (26.0)	40 (47.1)	416 (25.0)	
Missing	7	0	7	
Resectability				
Primary resectable	429 (24.5)	6 (7.1)	423 (25.4)	<.001^d^
Borderline resectable	856 (48.9)	43 (50.6)	813 (48.8)	
Locally advanced	465 (26.6)	36 (42.4)	429 (25.8)	
Missing	8	0	8	
Tumor location				
Pancreatic head	1276 (72.6)	71 (83.5)	1205 (72.1)	.02^d^
Pancreatic body or tail	481 (27.4)	14 (16.5)	467 (27.9)	
Missing	1	0	1	
Tumor size, mm				
≤20	385 (22.2)	9 (11.3)	376 (22.7)	.005^d^
21-40	1095 (63.1)	51 (63.8)	1044 (63.1)	
>40	255 (14.7)	20 (25.0)	235 (14.2)	
Missing	23	5	18	
cT stage				.10^d^
T1/T2	425 (24.2)	27 (31.8)	398 (23.8)	
T3/T4	1332 (75.8)	58 (68.2)	1274 (76.2)	
Missing	1	0	1	
CA 19-9, U/mL				
Median (IQR)	183 (46-626)	105 (31-475)	185 (48-633)	.11^e^
<37	317 (18.0)	16 (18.8)	301 (18.0)	<.001^d^
≥37 to <150	342 (19.5)	17 (20.0)	325 (19.4)	
≥150 to <500	360 (20.5)	8 (9.4)	352 (21.0)	
≥500 to <1000	156 (8.9)	6 (7.1)	150 (9.0)	
≥1000	259 (14.7)	8 (9.4)	251 (15.0)	
Missing	324 (18.4)	30 (35.3)	294 (17.6)	
CEA				
Median (IQR), ng/mL	3.1 (2.1-5.3)	2.6 (2.1-4.4)	3.2 (2.1-5.4)	.41^e^
Normal	633 (36.0)	21 (24.7)	612 (36.6)	<.001^f^
>5 to ≤20	204 (11.6)	2 (2.4)	202 (12.1)	
>20	34 (1.9)	2 (2.4)	32 (1.9)	
Missing	887 (50.5)	60 (70.6)	827 (49.4)	

Abbreviations: ASA-PS, American Society of Anesthesiologists Performance Status; CA 19-9, carbohydrate antigen 19-9; CEA, carcinoembryonic antigen; pCR, pathological complete response.

SI conversion factor: To convert CEA to micrograms per liter, multiply by 1.

^a^ Data are presented as number (percentage) of patients unless otherwise indicated. Categorical data with missing data for 2% or more of patients were included in a separate category and were therefore included in the overall proportions and in the test of association. Otherwise, for missing data less than 2%, the data are shown but were not included in the hypothesis tests. See eAppendix 4 in Supplement 1 for the presentation of these data using row percentages.

^b^ Comparison between patients with or without pCR.

^c^ Welch independent 2-sample *t* test.

^d^ χ^2^ test.

^e^ Wilcoxon rank sum test.

^f^ Fisher exact test.

### Preoperative Therapy and Disease Response

Most patients were treated with preoperative (modified) leucovorin calcium (folinic acid), fluorouracil, irinotecan hydrochloride, and oxaliplatin ([m]FOLFIRINOX) (n = 797 [45.3%]) or gemcitabine and nab-paclitaxel (n = 501 [28.5%]). Patients with pCR were more frequently treated with preoperative (m)FOLFIRINOX compared with patients without pCR (n = 50 of 85 [58.8%] vs 747 of 1673 [44.7%]; *P* < .001). Concomitant radiotherapy was administered in 872 patients (50.0%). The rate of radiation therapy was higher among patients with pCR compared with patients without pCR (n = 68 of 85 [80.0%] vs 804 of 1658 [48.5%]; *P* < .001). See Table 2 for details regarding preoperative therapy and response evaluation.

**Table 2.** Preoperative Therapy and Disease Response^a^

Variable	Patients, No. (%)			*P* value^b^
	Overall cohort (N = 1758)	pCR (n = 85)	No pCR (n = 1673)	
**Preoperative therapy**				
Chemotherapy				
(m)FOLFIRINOX	797 (45.3)	50 (58.8)	747 (44.7)	<.001^c^
Gemcitabine and nab-paclitaxel	501 (28.5)	11 (12.9)	490 (29.3)	
Gemcitabine–S-1	100 (5.7)	1 (1.2)	99 (5.9)	
Gemcitabine-oxaliplatin	101 (5.7)	2 (2.4)	99 (5.9)	
Other multiagent regimens	90 (5.1)	9 (10.6)	81 (4.8)	
Gemcitabine	97 (5.5)	7 (8.2)	90 (5.4)	
S-1	53 (3.0)	2 (2.4)	51 (3.0)	
Other single-agent regimens	19 (1.1)	3 (3.5)	16 (1.0)	
Second-line chemotherapy	216 (12.3)	13 (15.3)	203 (12.1)	.55^c^
Missing	74 (4.2)	2 (2.4)	72 (4.3)	
Chemotherapy dose reduction	250 (14.2)	6 (7.1)	244 (14.6)	<.001^d^
Missing	660 (37.5)	57 (67.1)	603 (36.0)	
Radiotherapy				
No	871 (50.0)	17 (20.0)	854 (51.5)	<.001^d^
Yes, conventional radiotherapy	462 (26.5)	37 (43.5)	425 (25.6)	
Yes, SBRT	410 (23.5)	31 (36.5)	379 (22.9)	
Missing	15	0	15	
Preoperative therapy duration, mo				
<4	475 (27.0)	16 (18.8)	459 (27.4)	<.001^c^
≥4 to <6	469 (26.7)	16 (18.8)	453 (27.1)	
≥6 to <12	637 (36.2)	34 (40.0)	603 (36.0)	
≥12	118 (6.7)	16 (18.8)	102 (6.1)	
Missing	59 (3.4)	3 (3.5)	56 (3.3)	
**Response evaluation**				
RECIST				
Complete response	14 (0.8)	4 (4.7)	10 (0.6)	<.001^c^
Partial response	506 (29.1)	61 (71.8)	445 (26.9)	
Stable disease	1194 (68.7)	19 (22.4)	1175 (71.0)	
Progressive disease	25 (1.4)	1 (1.2)	24 (1.5)	
Missing	19	0	19	
CA 19-9				
Median (IQR), U/mL	36 (15-92)	19 (13-35)	37 (15-95)	.003^e^
Normal	773 (44.0)	43 (50.6)	730 (43.6)	<.001^c^
≥37 to <150 U/mL	493 (28.0)	7 (8.2)	486 (29.0)	
≥150 to <500 U/mL	170 (9.7)	3 (3.5)	167 (10.0)	
≥500 to <1000 U/mL	37 (2.1)	1 (1.2)	36 (2.2)	
≥1000 U/mL	40 (2.3)	0	40 (2.4)	
Missing	245 (13.9)	31 (36.5)	214 (12.8)	
CA 19-9 patterns				
Normal to normal	281 (16.0)	12 (14.1)	269 (16.1)	<.001^c^
Normal to elevated	17 (1.0)	1 (1.2)	16 (1.0)	
Elevated to normal	415 (23.6)	23 (27.1)	392 (23.4)	
Elevated to elevated	655 (37.3)	10 (11.8)	645 (38.6)	
Missing	390 (22.2)	39 (45.9)	351 (21.0)	
Relative CA 19-9 change				
No change or increased	93 (5.3)	2 (2.4)	91 (5.4)	<.001^c^
Decreased <87%	538 (30.6)	9 (10.6)	529 (31.6)	
Decreased ≥87%	439 (25.0)	22 (25.9)	417 (24.9)	
<37 U/mL at time of diagnosis	317 (18.0)	16 (18.8)	301 (18.0)	
Missing	371 (21.1)	36 (42.4)	335 (20.0)	
CEA				
Median (IQR), ng/mL	2.9 (2.0-4.6)	2.9 (1.8-4.2)	2.9 (2.0-4.6)	.85^e^
Normal	744 (42.3)	21 (24.7)	723 (43.2)	<.001^c^
>5 to ≤20 ng/mL	189 (10.8)	3 (3.5)	186 (11.1)	
>20 ng/mL	20 (1.1)	0	20 (1.2)	
Missing	805 (45.8)	61 (71.8)	744 (44.5)	
CEA patterns				
Normal to normal	520 (29.6)	13 (15.3)	507 (30.3)	<.001^c^
Normal to elevated	53 (3.0)	1 (1.2)	52 (3.1)	
Elevated to normal	100 (5.7)	2 (2.4)	98 (5.9)	
Elevated to elevated	110 (6.3)	0	110 (6.6)	
Missing	975 (55.5)	69 (81.2)	906 (54.2)	

Abbreviations: CA 19-9, carbohydrate antigen 19-9; CEA, carcinoembryonic antigen; (m)FOLFIRINOX, (modified) leucovorin calcium (folinic acid), fluorouracil, irinotecan hydrochloride, and oxaliplatin; pCR, pathological complete response; RECIST, Response Evaluation Criteria in Solid Tumors; SBRT, stereotactic body radiotherapy.

SI conversion factor: To convert CEA to micrograms per liter, multiply by 1.

^a^ Data are presented as number (percentage) of patients unless otherwise indicated. Categorical data with missing data for 2% or more of patients were included in a separate category and were therefore included in the overall proportions and in the test of association. Otherwise, for missing less than 2%, the data are shown but were not included in the hypothesis tests. See eAppendix 4 in Supplement 1 for the presentation of these data using row percentages.

^b^ Comparison between patients with or without pCR.

^c^ Fisher exact test.

^d^ χ^2^ test.

^e^ Wilcoxon rank sum test.

### Surgery Outcome

Pancreatoduodenectomy was the most commonly performed surgical procedure (n = 1262 [71.8%]). An extended resection was performed in 696 patients (39.6%). The rates of vascular resection among patients with a primary resectable, borderline resectable, and locally advanced tumor were 16.1% (n = 69 of 429), 39.6% (n = 339 of 855 [missing n = 1]), and 44.3% (n = 206 of 465), respectively. The 90-day mortality was 3.2% (n = 55). See eAppendix 2 in Supplement 1 for further details on surgical procedures and outcome and eAppendix 3 in Supplement 1 for histopathological outcomes. Patients with pCR received adjuvant chemotherapy (n = 30 of 85 [35.3%]) less frequently than patients without pCR (n = 1120 of 1673 [66.9%]) (*P* < .001).

### Oncologic Outcome

Follow-up data were available from 1757 of the 1758 patients, of whom 817 patients (46.5%) died. The median (IQR) follow-up time was 19 (11-33) months. The median OS from the total cohort was 33 months (95% CI, 31-37 months) (Figure, A). The median OS outcomes of patients with a primary resectable, borderline resectable, or locally advanced tumor were 43 (95% CI, 36-49), 31 (95% CI, 27-35), and 31 (95% CI, 26-36) months (*P* = .19), respectively. The median OS was shorter in patients who underwent a vascular resection compared with no vascular resection: 28 (95% CI, 26-31) vs 38 (95% CI, 33-43) months (*P* < .001), respectively.

**Figure.**
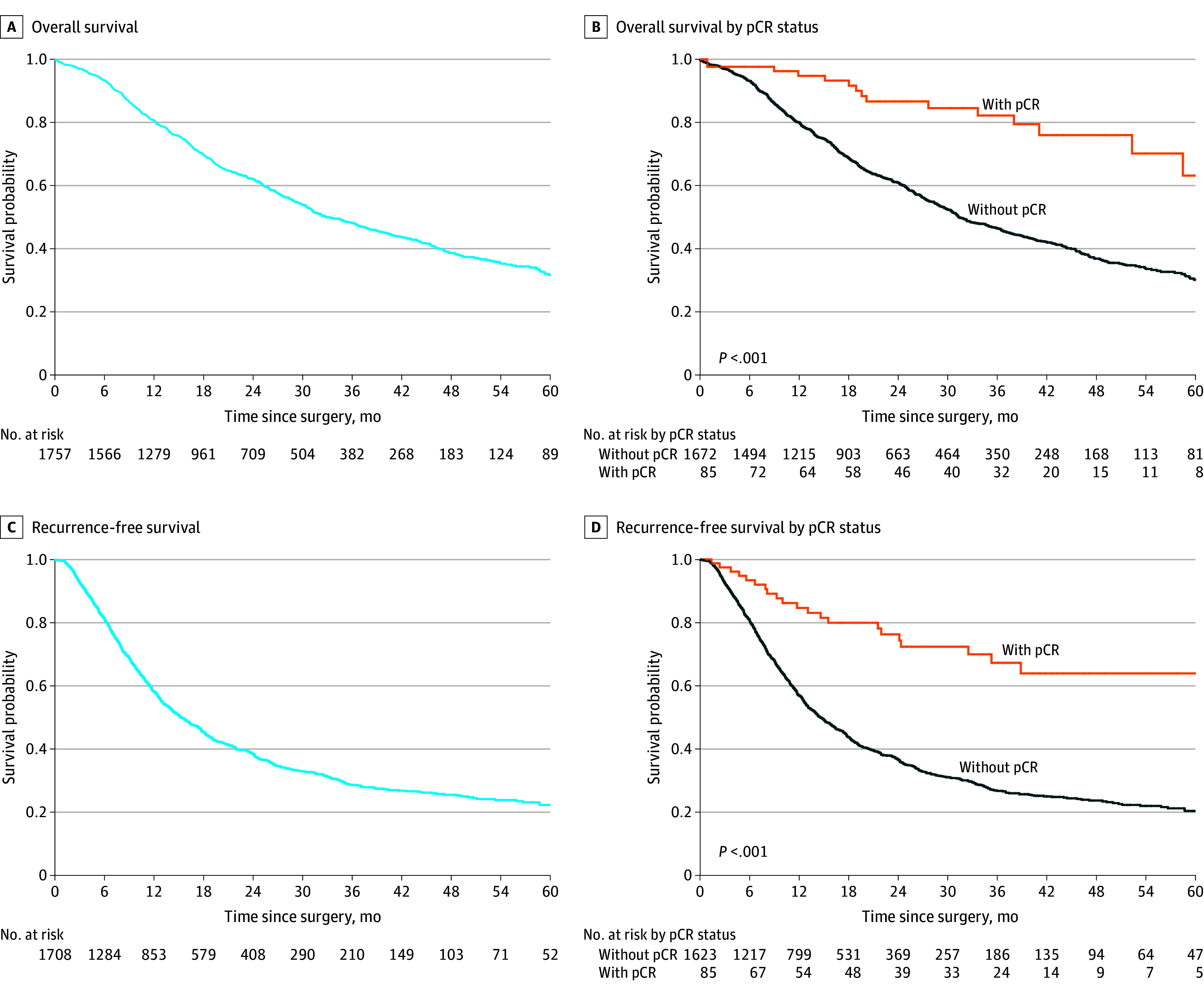
Kaplan-Meier Survival Curves pCR indicates pathological complete response.

When comparing patients with and without pCR, the median OS was not reached in the pCR group, whereas the median OS was 31 (95% CI, 30-35) months in patients without pCR (*P* < .001). In patients with pCR, the 1-, 3-, and 5-year OS rates were 95% (95% CI, 90%-100%), 82% (95% CI, 73%-93%), and 63% (95% CI, 47%-86%), respectively. In patients without pCR, the 1-, 3-, and 5-year OS rates were 80% (95% CI, 78%-82%), 46% (95% CI, 44%-49%), and 30% (95% CI, 27%-34%), respectively (Figure, B). See eAppendix 5 in Supplement 1 for OS outcomes measured from the start of preoperative chemotherapy.

For the analysis on RFS, 50 patients had missing data on recurrence status (n = 18) and/or date of disease recurrence (n = 33). Therefore, these patients were excluded from this analysis. Among the 1708 patients with data about disease recurrence, 1036 (60.7%) developed disease recurrence. In the overall study cohort, the median RFS was 16 months (95% CI, 14-17 months) (Figure, C). The median RFS was not reached in the patients with pCR, whereas the median RFS was 15 months (95% CI, 14-16 months) in patients without pCR (*P* < .001) (Figure, D). See eAppendix 6 in Supplement 1 for the comparison of recurrence location between patients with or without pCR. After adjustment for potential confounders, pCR was associated with prolonged OS (HR, 0.46; 95% CI, 0.26-0.83). See Table 3 for the Cox proportional hazards regression analysis.

**Table 3.** Cox Proportional Hazards Regression Model for Estimating Associations Between Risk Factors and Mortality

Variable	No. of patients (N = 1677)	Univariable analysis		Multivariable analysis	
		HR (95% CI)	*P* value	HR (95% CI)	*P* value
Gender					
Female	842	1 [Reference]	NA	1 [Reference]	NA
Male	835	1.11 (0.96-1.28)	.15	1.09 (0.94-1.25)	.27
ASA-PS					
I-II	1242	1 [Reference]	NA	NA	NA
III-IV	435	1.06 (0.90-1.25)	.49	NA	NA
Tumor location					
Body or tail	458	1 [Reference]	NA	NA	NA
Head	1219	0.94 (0.81-1.10)	.47	NA	NA
Tumor size at diagnosis, mm					
≤20	374	1 [Reference]	NA	1 [Reference]	NA
21-40	1059	1.31 (1.09-1.58)	.004	1.08 (0.88-1.33)	.46
>40	244	1.41 (1.11-1.80)	.006	0.79 (0.59-1.06)	.12
Resectability at diagnosis					
Primary resectable	417	1 [Reference]	NA	1 [Reference]	NA
Borderline resectable	819	1.14 (0.96-1.37)	.14	1.05 (0.86-1.28)	.62
Locally advanced	441	1.22 (1.00-1.49)	.05	1.16 (0.92-1.45)	.20
CA 19-9 at diagnosis, U/mL					
<37	305	1 [Reference]	NA	NA	NA
≥37 to <150	330	0.81 (0.64-1.02)	.07	NA	NA
≥150 to <500	340	1.09 (0.88-1.36)	.43	NA	NA
≥500 to <1000	146	0.95 (0.71-1.28)	.76	NA	NA
≥1000	249	1.21 (0.95-1.52)	.12	NA	NA
Missing	307	0.77 (0.60-0.98)	.03	NA	NA
Preoperative chemotherapy					
(m)FOLFIRINOX	764	1 [Reference]	NA	NA	NA
Other multiagent	754	1.06 (0.92-1.24)	.42	NA	NA
Single agent	159	1.03 (0.81-1.31)	.80	NA	NA
Preoperative radiotherapy					
None	835	1 [Reference]	NA	1 [Reference]	NA
Conventional radiotherapy	446	0.79 (0.66-0.94)	.008	0.97 (0.79-1.19)	.75
SBRT	396	1.09 (0.91-1.29)	.34	1.27 (1.04-1.56)	.02
Preoperative therapy duration, mo					
<4	452	1 [Reference]	NA	NA	NA
≥4 to <6	452	0.88 (0.73-1.07)	.20	NA	NA
≥6 to <12	609	1.03 (0.86-1.22)	.76	NA	NA
≥12	111	0.91 (0.67-1.25)	.56	NA	NA
Missing	53	0.90 (0.55-1.45)	.65	NA	NA
RECIST					
Stable	1147	1 [Reference]	NA	1 [Reference]	NA
Progressive disease	24	1.81 (1.12-2.93)	.02	1.55 (0.93-2.56)	.09
Partial or complete response	506	0.72 (0.61-0.85)	<.001	0.80 (0.67-0.96)	.02
CA 19-9 at restaging, U/mL					
≥37	696	1 [Reference]	NA	1 [Reference]	NA
<37 (normal[ization])	743	0.76 (0.65-0.88)	<.001	0.89 (0.76-1.04)	.15
Missing	238	0.82 (0.66-1.02)	.07	1.03 (0.80-1.33)	.80
Relative CA 19-9 change					
No change/increased	83	1 [Reference]	NA	NA	NA
Decreased <87%	513	0.84 (0.61-1.16)	.29	NA	NA
Decreased ≥87%	424	0.70 (0.51-0.98)	.04	NA	NA
<37 U/mL at diagnosis	305	0.79 (0.57-1.11)	.18	NA	NA
Missing	352	0.64 (0.46-0.90)	.01	NA	NA
pCR					
No	1597	1 [Reference]	NA	1 [Reference]	NA
Yes	80	0.31 (0.19-0.52)	<.001	0.46 (0.26-0.83)	.009
Residual disease					
R0	1225	1 [Reference]	NA	1 [Reference]	NA
R1-2	288	2.04 (1.72-2.41)	<.001	1.67 (1.38-2.01)	<.001
Unknown	164	1.28 (1.00-1.64)	.05	1.54 (1.10-2.15)	.01
Tumor size in histopathology, mm					
≤20	620	1 [Reference]	NA	1 [Reference]	NA
21-40	793	1.67 (1.42-1.97)	<.001	1.24 (1.03-1.49)	.03
>40	216	2.24 (1.79-2.79)	<.001	1.61 (1.24-2.09)	<.001
Missing	48	1.90 (1.28-2.81)	.001	1.20 (0.79-1.83)	.40
Lymphovascular invasion					
No	867	1 [Reference]	NA	1 [Reference]	NA
Yes	726	1.57 (1.36-1.82)	<.001	1.13 (0.95-1.33)	.16
Missing	84	1.33 (0.97-1.84)	.08	1.20 (0.77-1.86)	.42
Perineural invasion					
No	493	1 [Reference]	NA	1 [Reference]	NA
Yes	1130	1.79 (1.51-2.13)	<.001	1.22 (1.01-1.48)	.04
Missing	54	1.31 (0.85-2.01)	.22	0.93 (0.52-1.68)	.81
Tumor differentiation					
Gx	178	1 [Reference]	NA	1 [Reference]	NA
G1-G2	1030	1.16 (0.90-1.49)	.25	1.09 (0.83-1.44)	.54
G3-G4	313	1.87 (1.42-2.47)	<.001	1.60 (1.19-2.16)	.002
Missing	156	1.32 (0.94-1.84)	.11	1.35 (0.94-1.94)	.10
Lymph node status					
ypN0	895	1 [Reference]	NA	1 [Reference]	NA
ypN1-2	782	1.90 (1.64-2.19)	<.001	1.54 (1.30-1.81)	<.001
Metastatic disease					
M0	1191	1 [Reference]	NA	1 [Reference]	NA
M1	40	1.59 (1.04-2.43)	.03	1.26 (0.82-1.94)	.30
Mx	252	0.81 (0.65-1.01)	.06	1.07 (0.81-1.41)	.63
Missing	194	0.77 (0.61-0.99)	.04	0.68 (0.51-0.91)	.008
Major morbidity					
No	1160	1 [Reference]	NA	1 [Reference]	
Yes	250	1.43 (1.19-1.73)	<.001	1.28 (1.05-1.56)	.01
Missing	267	0.87 (0.71-1.07)	.19	0.65 (0.48-0.88)	.005
Adjuvant chemotherapy					
No	430	1 [Reference]	NA	1 [Reference]	NA
Yes	1098	0.60 (0.51-0.70)	<.001	0.50 (0.42-0.59)	<.001
Missing	149	0.79 (0.59-1.06)	.12	0.69 (0.50-0.95)	.02

Abbreviations: ASA-PS, American Society of Anesthesiologists Performance Status; CA 19-9, carbohydrate antigen 19-9; HR, hazard ratio; (m)FOLFIRINOX, (modified) leucovorin calcium (folinic acid), fluorouracil, irinotecan hydrochloride, and oxaliplatin; NA, not applicable; pCR, pathological complete response; RECIST, Response Evaluation Criteria in Solid Tumors; SBRT, stereotactic body radiotherapy.

### Factors Associated With pCR

Tumors located in the pancreatic head (OR, 2.51; 95% CI, 1.25-5.06), tumor size greater than 40 mm (vs ≤20 mm) on cross-sectional imaging at diagnosis (OR, 2.58; 95% CI, 1.03-6.48), conventional radiotherapy (vs no radiotherapy) (OR, 2.03; 95% CI, 1.00-4.10), stereotactic body radiation therapy (SBRT) (vs no radiotherapy) (OR, 8.91; 95% CI, 4.17-19.05), partial or complete radiologic response (vs stable disease) (OR, 13.00; 95% CI, 7.02-24.08), and serum CA 19-9 normal(ization) (vs CA 19-9 ≥37 U/mL) (OR, 3.76; 95% CI, 1.79-7.89) were associated with pCR. In contrast, preoperative multiagent chemotherapy other than (m)FOLFIRINOX (vs [m]FOLFIRINOX) (OR, 0.48; 95% CI, 0.26-0.87) was associated with not achieving pCR. See Table 4 for the logistic regression analysis.

**Table 4.** Logistic Regression Model for Estimating Associations Between Risk Factors and Pathological Complete Response

Variable	No. of patients (N = 1695)	Univariable analysis		Multivariable analysis	
		OR (95% CI)	*P* value	OR (95% CI)	*P* value
Gender					
Female	850	1 [Reference]	NA	NA	NA
Male	845	1.12 (0.71-1.75)	.63	NA	NA
Tumor location					
Body or tail	462	1 [Reference]	NA	1 [Reference]	NA
Head	1233	1.98 (1.08-3.63)	.03	2.51 (1.25-5.06)	.01
Tumor size at diagnosis, mm					
≤20	376	1 [Reference]	NA	1 [Reference]	NA
21-40	1073	2.03 (0.99-4.17)	.05	1.52 (0.69-3.33)	.29
>40	246	3.61 (1.62-8.06)	.002	2.58 (1.03-6.48)	.04
Resectability at diagnosis					
Primary resectable	424	1 [Reference]	NA	1 [Reference]	NA
Borderline resectable	827	3.54 (1.49-8.42)	.004	1.63 (0.60-4.40)	.34
Locally advanced	444	5.78 (2.40-13.91)	<.001	2.31 (0.84-6.36)	.10
CA 19-9 at diagnosis, U/mL					
<37	307	1 [Reference]	NA	NA	NA
≥37 to <150	331	0.99 (0.48-2.04)	.98	NA	NA
≥150 to <500	348	0.40 (0.16-0.99)	.05	NA	NA
≥500 to <1000	148	0.82 (0.31-2.16)	.69	NA	NA
≥1000	252	0.64 (0.27-1.53)	.31	NA	NA
Missing	309	1.94 (1.01-3.71)	.05	NA	NA
Preoperative chemotherapy					
(m)FOLFIRINOX	771	1 [Reference]	NA	1 [Reference]	NA
Other multiagent	765	0.38 (0.22-0.64)	<.001	0.48 (0.26-0.87)	.02
Single agent	159	1.20 (0.62-2.32)	.58	2.42 (0.99-5.92)	.05
Preoperative radiotherapy					
None	845	1 [Reference]	NA	1 [Reference]	NA
Conventional radiotherapy	448	3.75 (2.06-6.83)	<.001	2.03 (1.00-4.10)	.05
SBRT	402	4.07 (2.22-7.45)	<.001	8.91 (4.17-19.05)	<.001
Preoperative therapy duration, mo					
<4	456	1 [Reference]	NA	1 [Reference]	NA
≥4 to <6	455	1.15 (0.55-2.39)	.71	0.59 (0.24-1.46)	.25
≥6 to <12	616	1.67 (0.88-3.18)	.12	0.63 (0.27-2.45)	.27
≥12	113	5.21 (2.46-11.03)	<.001	2.13 (0.83-5.43)	.12
Missing	55	1.82 (0.51-6.55)	.36	0.99 (0.23-4.23)	.98
RECIST					
Stable disease	1165	1 [Reference]	NA	1 [Reference]	NA
Progressive disease	24	2.94 (0.37-23.00)	.31	1.84 (0.20-17.30)	.59
Partial or complete response	506	9.43 (5.45-16.31)	<.001	13.00 (7.02-24.08)	<.001
CA 19-9 at restaging, U/mL					
≥37	709	1 [Reference]	NA	1 [Reference]	NA
<37 (normal[ization])	748	3.59 (1.82-7.04)	<.001	3.76 (1.79-7.89)	<.001
Missing	238	8.80 (4.32-17.92)	<.001	10.89 (4.73-25.06)	<.001
Relative CA 19-9 change					
No change or increased	86	1 [Reference]	NA	NA	NA
Decreased <87%	521	0.74 (0.16-3.48)	.70	NA	NA
Decreased ≥87%	427	2.17 (0.50-9.44)	.30	NA	NA
<37 U/mL at diagnosis	307	2.16 (0.48-9.62)	.31	NA	NA
Missing	354	4.32 (1.02-18.36)	.05	NA	NA

Abbreviations: CA 19-9, carbohydrate antigen 19-9; (m)FOLFIRINOX, (modified) leucovorin calcium (folinic acid), fluorouracil, irinotecan hydrochloride, and oxaliplatin; NA, not applicable; OR, odds ratio; RECIST, Response Evaluation Criteria in Solid Tumors; SBRT, stereotactic body radiotherapy.

## Discussion

This retrospective, international cohort study of 1758 patients who underwent resection of pancreatic adenocarcinoma after preoperative chemo(radio)therapy demonstrated that pCR occurs in 4.8% of patients and is associated with better OS compared with patients without pCR. This finding is illustrated by the 3-fold higher 5-year RFS (64% vs 20%) and doubled 5-year OS (63% vs 30%) compared with patients without pCR. Factors associated with pCR included a tumor located in the pancreatic head, larger tumors, (m)FOLFIRINOX chemotherapy compared with other multiagent regimens, preoperative conventional radiotherapy and SBRT, partial or complete radiologic response, and normal(ized) serum CA 19-9 at restaging.

The 4.8% rate of pCR in the current international study is somewhat higher than the 4% reported by a systematic review comprising 27 prospective studies including 1129 patients with localized pancreatic adenocarcinoma.^13^ This finding could be explained by the more recent study period of our study because preoperative (m)FOLFIRINOX and modern radiotherapeutic modalities are probably more often used in recent years. Both (m)FOLFIRINOX and radiotherapy were associated with pCR after adjustment, whereby SBRT had a stronger association compared with conventional radiotherapy. This hypothesis is strengthened by the increasing incidence of pCR in the period 2004 to 2016, according to the National Cancer Database.^14^

This is the first study, to our knowledge, investigating the association between pCR and preoperative treatment strategies (ie, chemotherapy regimen and radiotherapeutic modalities) and serum CA 19-9. Previous reports have queried databases with small sample sizes or large databases with limited information about preoperative therapy and clinicopathological characteristics.^10,11,14,16,20,21,40^ Cloyd et al^14^ studied the National Cancer Database (pCR in 244 of 7902 patients [3.0%] diagnosed with localized pancreatic cancer [2004-2016]) and demonstrated that preoperative multiagent vs single-agent chemotherapy was not associated with pCR. In the current study, however, the odds of developing pCR were greater after (m)FOLFIRINOX compared with other multiagent chemotherapies. Remarkably, no difference was seen between (m)FOLFIRINOX and single-agent chemotherapy in our study. In contrast to the findings in our study, Cloyd et al^11^ demonstrated that the duration of preoperative therapy was associated with pCR. The absence of this association in the current study could be explained by probably a higher rate of modern, more potent preoperative therapies (eg, [m]FOLFIRINOX and SBRT). Some recent literature suggests the potential value of total neoadjuvant therapy (ie, chemotherapy followed by chemoradiotherapy) compared with chemotherapy followed by radiotherapy, chemoradiotherapy, or chemotherapy alone, possibly associated with higher rate of pCR and/or OS.^17,19,41^ In general, preoperative chemotherapy with radiation is associated with improved pathological outcomes (eg, higher rates of R0, negative lymph nodes, and tumor response) compared with chemotherapy alone, but this rarely translates into prolonged OS^42,43^ and might even be considered harmful when an arterial resection or divestment is needed during surgery.^43,44^ The lack of OS benefit was also seen in the current study, in which preoperative radiotherapy did not improve OS and SBRT was even independently associated with impaired OS. However, SBRT was associated with pCR. This finding seems conflicting because pCR is associated with longer OS (ie, median OS not reached; 63% 5-year OS), as reported by previous studies.^14,15,17^ This divergence suggests that pCR might not always reflect an optimal disease response and does not guarantee cure,^45^ illustrated by the 5-year RFS rate of 64% in this study. Nevertheless, the lower serum CA 19-9 level at restaging among patients with pCR and the association of normal(ized) serum CA 19-9 with pCR suggest that pCR represents both local and systemic disease responses in a substantial group of patients, leading to prolonged OS. Of note, the association of SBRT with shorter OS should be interpreted with caution because this finding is derived solely from patients who underwent a resection. Randomized clinical trials are necessary to determine the value of additional radiotherapy.^46^

The 5-year OS was doubled in patients with pCR compared with those without pCR in this study (63% vs 30%). Therefore, it is clear that pCR cannot be interpreted as synonymous with cure, also considering the 5-year RFS of 64%. Serum CA 19-9 response for prognostication and clinical decision-making in patients with pCR seems crucial because it might be a surrogate marker for the systemic disease response.^47,48^ Only 35% of patients with pCR received adjuvant chemotherapy compared with 67% of patients without pCR. Certain patients with pCR may benefit from additional systemic chemotherapy. However, the remaining micrometastases might have different genetic or molecular characteristics compared with the primary tumor, which might be responsible for other chemosensitivity or chemotherapy resistance.^49^ Better tumor markers are needed to detect the presence of remaining systemic disease.^46^

Reliable pathological assessment is a major concern. Recently, the International Study Group of Pancreatic Pathologists^12^ found moderate correlations of 0.66 and 0.71 for tumor regression grading among 23 world-leading pancreatic pathologists, using the College of American Pathologists and MD Anderson Cancer Center classification systems.^28^ Eight of 50 patients were classified as having pCR by at least 1 pathologist, but consensus was reached in none of the cases.^28^ Furthermore, variation exists among pathologists in the sampling strategy, varying from complete specimen sampling to macroscopy-based tumor sampling.^30^ Because pancreatic adenocarcinoma is characterized by irregular distribution of tumor cells embedded in stroma and fibrosis (ie, intratumor heterogeneity), vital tumor cells can be easily missed when the specimen is not fully sampled, particularly when the distance between vital cells further increases due to preoperative chemotherapy.^30^ These limitations affect the reliability of the diagnosis of pCR and could have contributed to the variability of pCR incidence per center in this study (median [IQR], 3.8% [0.3%-7.8%]). Unfortunately, the retrospective nature of the current study prohibited reliable data collection regarding the sampling strategies. Even though it is not unlikely that the pCR group in this study also contains patients with vital tumor cells left in the resected pancreas specimen, the associated prolonged OS suggests the presence of at least extensive tumor response.

### Strengths and Limitations

This study has several strengths. The major strength is the large number of patients originating from multiple countries and continents (see eAppendix 7 in Supplement 1 for region-specific data) and having detailed data on treatment and disease characteristics. Furthermore, most patients were treated with modern preoperative regimens, reflecting the current clinical practices. Of note, one-quarter of the included patients were diagnosed with a primary resectable tumor, possibly treated with neoadjuvant therapy in the setting of clinical trials because neoadjuvant therapy was generally not the standard of care during the study period.^50,51,52^

Future research should focus on improving tumor response scoring systems. Artificial intelligence models have the potential to accurately and objectively determine residual tumor burden.^53^ Such models need to be further developed and validated. For now, pancreatic pathologists should strive for a uniform strategy for sampling and response evaluation. Nevertheless, the patient does not die because of what the surgeon takes out but rather what is left behind. Therefore, there is an urgent need for better markers (eg, solid, liquid, and imaging based),^54,55,56^ allowing more adequate response evaluation, patient selection for surgery, and postoperative clinical decision-making for adjuvant therapy.^46^ Fluorodeoxyglucose positron emission tomography with computed tomography or magnetic resonance imaging is a promising tool to identify biological tumor response after preoperative therapy, including pCR. However, this method needs to be investigated in all-comers instead of solely surgical cohorts.^57,58^

The results of this study should be interpreted in light of some limitations. First, it was considered infeasible to perform a Cox proportional hazards regression analysis on OS within the cohort of patients with pCR because of the sample size and the limited number of events. Information about associated factors might elucidate in which patients pCR means a cure or requires adjuvant therapy and whether there is a difference in prognosis for pCR after preoperative chemotherapy with or without radiotherapy. Comparative subanalyses were underpowered by the small subgroups and small number of events. Second, a more detailed stratification for preoperative chemotherapy regimens was not feasible due to the number of patients with pCR. Third, a Cox proportional hazards regression analysis investigating potential factors associated with RFS was not performed because of heterogeneity in local follow-up strategies among centers. Fourth, information about patients’ race and presence of *BRCA* germline mutations were not collected or available, whereas these factors seem to be of relevance for the chance to achieve major pathological response.^59,60^ Fifth, the number of patients with R1 was relatively low (17%), which could be explained by different local protocols for which not all specimen surfaces were assessed. Sixth, serum bilirubin levels were not collected; therefore, serum CA 19-9 levels might be reactively elevated in some patients due to hyperbilirubinemia. Seventh, patients with pCR were included when preoperative pathology was suggestive of or conclusive for malignancy^22^ but without central review. Therefore, the diagnosis of (pancreatic) adenocarcinoma might not always have been certain.^61^

## Conclusions

In this international, observational, multicenter cohort study, pCR was found in 4.8% of patients with resected pancreatic adenocarcinoma after chemo(radio)therapy. Although pCR does not reflect cure, it is associated with better OS. Factors associated with pCR included preoperative chemotherapy regimens, radiation, and anatomical and biological disease response, which may have implications for treatment strategies. This finding should be confirmed in prospective studies because these factors may not universally apply to all patients with pancreatic adenocarcinoma, as illustrated by the association of SBRT with impaired OS, whereas SBRT was also associated with pCR.

## Notes

Supplement 1. **eAppendix 1.** Incidence of pCR per Center **eAppendix 2.** Surgical Details, Surgical Outcome, and Adjuvant Therapy **eAppendix 3.** Histopathology **eAppendix 4.** Clinicopathological and Treatment Characteristics (Row Percentages) **eAppendix 5.** Overall Survival From Starting Preoperative Chemotherapy **eAppendix 6.** Site of Disease Recurrence in Patients With pCR vs Without pCR **eAppendix 7.** Region-Specific Data

Supplement 2.
Data Sharing Statement
