# Supplementary material for: Pathological Complete Response in Patients With Resected Pancreatic Adenocarcinoma After Preoperative Chemotherapy
Source: JAMA Netw Open. 2024 Jun 18;7(6):e2417625. doi: 10.1001/jamanetworkopen.2024.17625 (PMC11185983; doi:10.1001/jamanetworkopen.2024.17625)
Supplement: Supplement 1. — eAppendix 1. Incidence of pCR per Center eAppendix 2. Surgical Details, Surgical Outcome, and Adjuvant Therapy eAppendix 3. Histopathology eAppendix 4. Clinicopathological and Treatment Characteristics (Row Percentages) eAppendix 5. Overall Survival From Starting Preoperative Chemotherapy eAppendix 6. Site of Disease Recurrence in Patients With pCR vs Without pCR eAppendix 7. Region-Specific Data [file jamanetwopen-e2417625-s001.pdf]

Supplementary Online Content

Stoop TF, Oba A, Wu YHA, et al. Pathological complete response in patients with resected pancreatic adenocarcinoma after preoperative chemotherapy. *JAMA Netw Open*. 2024;7(6):e2417625. doi:10.1001/jamanetworkopen.2024.17625

eAppendix 1. Incidence of pCR per Center

eAppendix 2. Surgical Details, Surgical Outcome, and Adjuvant Therapy

eAppendix 3. Histopathology

eAppendix 4. Clinicopathological and Treatment Characteristics (Row Percentages)

eAppendix 5. Survival

eAppendix 6. Recurrence Site in Patients With Disease Recurrence

eAppendix 7. Region-Specific Data

This supplementary material has been provided by the authors to give readers additional information about their work.

© 2024 Stoop TF et al. *JAMA Network Open.*

APPENDIX 1. Incidence of pCR per center

Center	Total number of patients (*n*)	Patients with pCR (*n*)	Incidence of pCR (%)
1	131	5	3.8
2	188	1	0.5
3	99	0	0
4	5	2	40.0
5	84	0	0
6	63	1	1.6
7	14	0	0
8	23	2	8.7
9	597	45	7.5
10	58	1	1.7
11	6	0	0
12	34	3	8.8
13	25	2	8.0
14	49	2	4.1
15	101	3	3.0
16	200	15	7.5
17	41	0	0
18	19	2	10.5
19	21	1	4.8

pCR, pathological complete response

© 2024 Stoop TF et al. *JAMA Network Open.*

APPENDIX 2. Surgical details, surgical outcome, and adjuvant therapy

	Patients, No. (%)			
	**Overall cohort**	**pCR**	**No pCR**	
**Variables**	***n* = 1758**	***n* = 85**	***n* = 1673**	***P* value[±]**
Pancreatectomy				
Pancreatoduodenectomy	1262 (71.8)	66 (77.6)	1196 (71.5)	.34[c]
Distal pancreatectomy	400 (22.8)	14 (16.5)	386 (23.1)	
Total pancreatectomy	96 (5.5)	5 (5.9)	91 (5.4)	
Extended resection	696 (39.6)	30 (35.3)	666 (39.8)	.40[b]
Vascular resection	615 (35.0)	21 (24.7)	594 (35.5)	**.04[b]**
None	1142 (65.0)	64 (75.3)	1078 (64.5)	**.05[b]**
Venous alone	476 (27.1)	20 (23.5)	456 (27.3)	
Arterial alone	135 (7.7)	1 (1.2)	134 (8.0)	
Venous + arterial	4 (0.2)	0 (0)	4 (0.2)	
Missing	1	0	1	
Multivisceral resection	128 (7.3)	11 (12.9)	117 (7.0)	**.04[b]**
Missing	1	0	1	
Major morbidity	259 (14.7)	11 (12.9)	248 (14.8)	.68[b]
Missing	271 (15.4)	11 (12.9)	260 (15.5)	
90-day mortality	55 (3.2)	2 (2.6)	53 (3.2)	>.999[c]
Missing	32	7	25	
Adjuvant chemotherapy	1150 (65.4)	30 (35.3)	1120 (66.9)	**<.001[b]**
Missing	157 (8.9)	6 (7.1)	151 (9.0)	

pCR, pathological complete response; ±, comparison between patients with or without pCR. *a*, Welch independent two-sample T-Test; *b*, Chi-square Test; *c*, Fisher's Exact Test; *d*, Wilcoxon Rank Sum Test.

NB. Categorical data with missing data for ≥2% of patients were included in a separate category and were therefore included in the overall proportions and in the test of association. Otherwise, for missing <2%, the data are shown but were not included in the hypothesis tests.

NB. See **Appendix 4** for the presentation of these data using row percentages.

© 2024 Stoop TF et al. *JAMA Network Open.*

APPENDIX 3. Histopathology

Variables	Patients, No. (%) Overall cohort *n* = 1758	pCR *n* = 85	No pCR *n* = 1673	*P* value[±]
Tumor size				
≤ 20 mm	646 (36.7)	72 (84.7)	574 (34.3)	**<.001[c]**
21 - 40 mm	830 (47.2)	6 (7.1)	824 (49.3)	
> 40 mm	225 (12.8)	4 (4.7)	221 (13.2)	
Missing	57 (3.2)	3 (3.5)	54 (3.2)	
ypT stage				
Tx/T0	94 (5.3)	83 (97.6)	11 (0.7)	**<.001[c]**
Tis	5 (0.3)	2 (2.4)	3 (0.2)	
T1	325 (18.5)	0 (0)	325 (19.4)	
T2	367 (20.9)	0 (0)	367 (21.9)	
T3	897 (51.0)	0 (0)	897 (53.6)	
T4	70 (4.0)	0 (0)	70 (4.2)	
ypN stage				
N0	936 (53.6)	83 (97.6)	853 (51.3)	**<.001[c]**
N1-2	811 (46.4)	2 (2.4)	809 (48.7)	
Missing	11	0	11	
ypM stage				
M0	1252 (71.2)	67 (78.8)	1185 (70.8)	**.03[c]**
M1	44 (2.5)	0 (0)	44 (2.6)	
Mx	265 (15.1)	15 (17.6)	250 (14.9)	
Missing	197 (11.2)	3 (3.5)	194 (11.6)	
Lymphovascular invasion	763 (43.4)	0 (0)	763 (45.6)	**<.001[c]**
Missing	94 (5.3)	12 (14.1)	82 (4.9)	
Perineural invasion	1177 (67.0)	0 (0)	1177 (70.4)	**<.001[c]**
Missing	63 (3.6)	12 (14.1)	51 (3.0)	
Differentiation				
Gx (not possible to determine)	190 (10.8)	60 (70.6)	130 (7.8)	**<.001[c]**
G1 (well differentiated)	241 (13.7)	0 (0)	241 (14.4)	
G2 (moderate differentiated)	831 (47.3)	0 (0)	831 (49.7)	
G3 (poorly differentiated)	320 (18.2)	0 (0)	320 (19.1)	
G4 (undifferentiated)	3 (0.2)	0 (0)	3 (0.2)	
Missing	173 (9.8)	25 (29.4)	148 (8.8)	
Residual disease				
R0	1267 (72.1)	79 (92.9)	1188 (71.0)	**<.001[c]**
R1	299 (17.0)	0 (0)	299 (17.9)	
R2	8 (0.5)	0 (0)	8 (0.5)	
Unknown/missing	184 (10.5)	6 (7.1)	178 (10.6)	

pCR, pathological complete response; *ml,* milliliters; ±, comparison between patients with or without pCR. *a*, Welch independent two-sample T-Test; *b*, Chi-square Test; *c*, Fisher's Exact Test; *d*, Wilcoxon Rank Sum Test.

NB. Categorical data with missing data for ≥2% of patients were included in a separate category and were therefore included in the overall proportions and in the test of association. Otherwise, for missing <2%, the data are shown but were not included in the hypothesis tests. NB. In some patients with pCR, a tumor size >0mm was registered. Because no vital tumor cells were detected, this suggests that the assumed 'former non-vital tumor tissue' is included in calculating the tumor size by the local pathologist. NB. In some patients with pCR, data about lymphovascular invasion, perineural invasion, tumor differentiation, and/or residual disease is missing. This data is handled as missing, although the absence of lymphovascular and perineural invasion, impossibility to determine differentiation, and no residual disease in the resection margins can be assumed. NB. See **Appendix 4** for the presentation of these data using row percentages.

© 2024 Stoop TF et al. *JAMA Network Open.*

APPENDIX 4. Clinicopathological and treatment characteristics (row percentages)

Variables	Patients, No. (%) Overall cohort *n* = 1758	pCR *n* = 85	No pCR *n* = 1673	*P* value±
**Baseline characteristics at diagnosis**				
Age (mean ± SD) (years)	64 (9)	62 (9)	64 (9)	**.01**[a]
Sex				
Female	879 (100)	41 (4.7)	838 (95.3)	.74[b]
Male	879 (100)	44 (5.0)	835 (95.0)	
ASA-PS				
I-II	1295 (100)	45 (3.5)	1250 (96.5)	**<.001**[b]
III-IV	456 (100)	40 (8.8)	416 (91.2)	
Missing	7	0	7	
Resectability				
Primary resectable	429 (100)	6 (1.4)	423 (98.6)	**<.001**[b]
Borderline resectable	856 (100)	43 (5.0)	813 (95.0)	
Locally advanced	465 (100)	36 (7.7)	429 (92.3)	
Missing	8	0	8	
Tumor location				
Pancreatic head	1276 (100)	71 (5.6)	1205 (94.4)	**.02**[b]
Pancreatic body/tail	481 (100)	14 (2.9)	467 (97.1)	
Missing	1	0	1	
Tumor size				
≤ 20 mm	385 (100)	9 (2.3)	376 (97.7)	**.005**[b]
21 - 40 mm	1095 (100)	51 (4.7)	1044 (95.3)	
> 40 mm	255 (100)	20 (7.8)	235 (92.2)	
Missing	23	5	18	
cT stage				.10[b]
T1/T2	425 (100)	27 (6.4)	398 (93.6)	
T3/T4	1332 (100)	58 (4.4)	1274 (95.6)	
Missing	1	0	1	
CA-19.9				
Median (IQR)	183 (46-626)	105 (31-475)	185 (48-633)	.11[d]
< 37 U/ml	317 (100)	16 (5.0)	301 (95.0)	**<.001**[b]
≥ 37 - < 150 U/ml	342 (100)	17 (5.0)	325 (95.0)	
≥ 150 - < 500 U/ml	360 (100)	8 (2.2)	352 (97.8)	
≥ 500 - < 1000 U/ml	156 (100)	6 (3.8)	150 (96.2)	
≥ 1000 U/ml	259 (100)	8 (3.1)	251 (96.9)	
Missing	324 (100)	30 (9.3)	294 (90.7)	
CEA				
Median (IQR)	3.1 (2.1-5.3)	2.6 (2.1-4.4)	3.2 (2.1-5.4)	.41[d]
Normal	633 (100)	21 (3.3)	612 (96.7)	**<.001**[c]
> 5 - ≤ 20 ng/ml	204 (100)	2 (1.0)	202 (99.0)	
> 20 ng/ml	34 (100)	2 (5.9)	32 (94.1)	
Missing	887 (100)	60 (70.6)	827 (93.2)	
**Preoperative therapy**				
Chemotherapy*				
(m)FOLFIRINOX	797 (100)	50 (6.3)	747 (93.7)	**<.001**[c]
Gemcitabine-nab-paclitaxel	501 (100)	11 (2.2)	490 (97.8)	
Gemcitabine-S1	100 (100)	1 (1.0)	99 (99.0)	

© 2024 Stoop TF et al. *JAMA Network Open.*

Gemcitabine-oxaliplatin	101 (100)	2 (2.0)	99 (98.0)	
Other multi-agent regimens	90 (100)	9 (10.0)	81 (90.0)	
Gemcitabine	97 (100)	7 (7.2)	90 (92.8)	
S1	53 (100)	2 (3.8)	51 (96.2)	
Other single-agent regimens	19 (100)	3 (15.8)	16 (84.2)	
Second-line chemotherapy				
No	1468 (100)	70 (4.8)	1398 (95.2)	.55[c]
Yes	216 (100)	13 (6.0)	203 (94.0)	
Missing	74 (100)	2 (2.7)	72 (97.3)	
Chemotherapy dose reduction				
No	848 (100)	22 (2.6)	826 (97.4)	**<.001[b]**
Yes	250 (100)	6 (2.4)	244 (97.6)	
Missing	660 (100)	57 (8.6)	603 (91.4)	
Radiotherapy				
No	871 (100)	17 (2.0)	854 (98.0)	**<.001[b]**
Yes, conventional radiotherapy	462 (100)	37 (8.0)	425 (92.0)	
Yes, SBRT	410 (100)	31 (7.6)	379 (92.4)	
Missing	15	0	15	
Preoperative therapy duration				
< 4 months	475 (100)	16 (3.4)	459 (96.6)	**<.001[c]**
≥ 4 to < 6 months	469 (100)	16 (3.4)	453 (96.6)	
≥ 6 to < 12 months	637 (100)	34 (5.3)	603 (94.7)	
≥ 12 months	118 (100)	16 (13.6)	102 (86.4)	
Missing	59 (100)	3 (5.1)	56 (94.9)	
**Response evaluation**				
RECIST				
Complete response	14 (100)	4 (28.6)	10 (71.4)	**<.001[c]**
Partial response	506 (100)	61 (12.1)	445 (87.9)	
Stable disease	1194 (100)	19 (1.6)	1175 (98.4)	
Progressive disease	25 (100)	1 (4.0)	24 (96.0)	
Missing	19	0	19	
CA-19.9				
Median (IQR)	36 (15-92)	19 (13-35)	37 (15-95)	**.003[d]**
Normal	773 (100)	43 (5.6)	730 (94.4)	**<.001[c]**
≥ 37 - < 150 U/ml	493 (100)	7 (1.4)	486 (98.6)	
≥ 150 - < 500 U/ml	170 (100)	3 (1.8)	167 (98.2)	
≥ 500 - < 1000 U/ml	37 (100)	1 (2.7)	36 (97.3)	
≥ 1000 U/ml	40 (100)	0 (0)	40 (100)	
Missing	245 (100)	31 (12.7)	214 (87.3)	
CA-19.9 patterns				
Normal to normal	281 (100)	12 (4.3)	269 (95.7)	**<.001[c]**
Normal to elevated	17 (100)	1 (5.9)	16 (94.1)	
Elevated to normal	415 (100)	23 (5.5)	392 (94.5)	
Elevated to elevated	655 (100)	10 (1.5)	645 (98.5)	
Missing	390 (100)	39 (10.0)	351 (90.0)	
Relative CA-19.9 change				
No change/increased	93 (100)	2 (2.2)	91 (97.8)	**<.001[c]**
Decreased < 87%	538 (100)	9 (1.7)	529 (98.3)	
Decreased ≥ 87%	439 (100)	22 (5.0)	417 (95.0)	

© 2024 Stoop TF et al. *JAMA Network Open.*

< 37 U/ml at time of diagnosis	317 (100)	16 (5.0)	301 (95.0)	
Missing	371 (100)	36 (9.7)	335 (90.3)	
CEA				
Median (IQR)	2.9 (2.0-4.6)	2.9 (1.8-4.2)	2.9 (2.0-4.6)	.85[d]
Normal	744 (100)	21 (2.8)	723 (97.2)	**<.001[c]**
> 5 - ≤ 20 ng/ml	189 (100)	3 (1.6)	186 (98.4)	
> 20 ng/ml	20 (100)	0 (0)	20 (100)	
Missing	805 (100)	61 (7.6)	744 (92.4)	
CEA patterns				
Normal to normal	520 (100)	13 (2.5)	507 (97.5)	**<.001[c]**
Normal to elevated	53 (100)	1 (1.9)	52 (98.1)	
Elevated to normal	100 (100)	2 (2.0)	98 (98.0)	
Elevated to elevated	110 (100)	0 (0)	110 (100)	
Missing	975 (100)	69 (7.1)	906 (92.9)	
**Surgery details, surgical outcome, and adjuvant therapy**				
Pancreatectomy				
Pancreatoduodenectomy	1262 (100)	66 (5.2)	1196 (94.8)	.34[c]
Distal pancreatectomy	400 (100)	14 (3.5)	386 (96.5)	
Total pancreatectomy	96 (100)	5 (5.2)	91 (98.4)	
Extended resection	696 (100)	30 (4.3)	666 (95.7)	.40[b]
Vascular resection	615 (100)	21 (3.4)	594 (96.6)	**.04[b]**
None	1142 (100)	64 (5.6)	1078 (94.4)	**.05[b]**
Venous alone	476 (100)	20 (4.2)	456 (95.8)	
Arterial alone	135 (100)	1 (0.7)	134 (99.3)	
Venous + arterial	4 (100)	0 (0)	4 (100)	
Missing	1	0	1	
Multivisceral resection	128 (100)	11 (8.6)	117 (91.4)	**.04[b]**
Missing	1	0	1	
Major morbidity				
No	1228 (100)	63 (5.1)	1165 (94.9)	.68[b]
Yes	259 (100)	11 (4.2)	248 (95.8)	
Missing	271 (100)	11 (4.1)	260 (95.9)	
90-day mortality				
No	1671 (100)	76 (4.5)	1595 (95.5)	>.999[c]
Yes	55 (3.2)	2 (3.6)	53 (96.4)	
Missing	32	7	25	
Adjuvant chemotherapy				
No	451 (100)	49 (10.9)	402 (89.1)	**<.001[b]**
Yes	1150 (100)	30 (2.6)	1120 (97.4)	
Missing	157 (100)	6 (3.8)	151 (96.2)	
**Histopathology**				
Tumor size				
≤ 20 mm	646 (100)	72 (11.1)	574 (88.9)	**<.001[c]**
21 - 40 mm	830 (100)	6 (0.7)	824 (99.3)	
> 40 mm	225 (100)	4 (1.8)	221 (98.2)	
Missing	57 (100)	3 (5.3)	54 (94.7)	
ypT stage				
Tx/T0	94 (100)	83 (88.3)	11 (11.7)	**<.001[c]**
Tis	5 (100)	2 (40.0)	3 (60.0)	

© 2024 Stoop TF et al. *JAMA Network Open.*

T1	325 (100)	0 (0)	325 (100)	
T2	367 (100)	0 (0)	367 (100)	
T3	897 (100)	0 (0)	897 (100)	
T4	70 (100)	0 (0)	70 (100)	
ypN stage				
N0	936 (100)	83 (8.9)	853 (91.1)	**<.001[c]**
N1-2	811 (100)	2 (0.2)	809 (99.8)	
Missing	11	0	11	
ypM stage				
M0	1252 (100)	67 (5.4)	1185 (94.6)	**.03[c]**
M1	44 (100)	0 (0)	44 (100)	
Mx	265 (100)	15 (5.7)	250 (94.3)	
Missing	197 (100)	3 (1.5)	194 (98.5)	
Lymphovascular invasion				
No	901 (100)	73 (8.1)	828 (91.9)	**<.001[c]**
Yes	763 (100)	0 (0)	763 (100)	
Missing	94 (100)	12 (12.8)	82 (87.2)	
Perineural invasion				
No	518 (100)	73 (14.1)	445 (85.9)	**<.001[c]**
Yes	1177 (100)	0 (0)	1177 (100)	
Missing	63 (100)	12 (19.0)	51 (81.0)	
Differentiation				
Gx (not possible to determine)	190 (100)	60 (31.6)	130 (68.4)	**<.001[c]**
G1 (well differentiated)	241 (100)	0 (0)	241 (100)	
G2 (moderate differentiated)	831 (100)	0 (0)	831 (100)	
G3 (poorly differentiated)	320 (100)	0 (0)	320 (100)	
G4 (undifferentiated)	3 (100)	0 (0)	3 (100)	
Missing	173 (100)	25 (14.5)	148 (85.5)	
Residual disease				
R0	1267 (100)	79 (6.2)	1188 (93.8)	**<.001[c]**
R1	299 (100)	0 (0)	299 (100)	
R2	8 (100)	0 (0)	8 (100)	
Unknown/missing	184 (100)	6 (3.3)	178 (96.7)	

pCR, pathological complete response; *SD,* standard deviation; *ASA-PS,* American Society of Anesthesiologists Performance Status; *mm,* millimeters; *CA-19.9,* carbohydrate 19.9; *U,* units; *ml,* milliliters; *CEA,* carcinoembryonic antigen; *ng,* nanogram; *SBRT,* stereotactic body radiotherapy; ±, comparison between patients with or without pCR. *a*, Welch independent two-sample T-Test; *b*, Chi-square Test; *c*, Fisher's Exact Test; *d*, Wilcoxon Rank Sum Test.

Categorical data with missing data for ≥2% of patients were included in a separate category and were therefore included in the overall proportions and in the test of association. Otherwise, for missing <2%, the data are shown but were not included in the hypothesis tests.

© 2024 Stoop TF et al. *JAMA Network Open.*

APPENDIX 5. Survival

APPENDIX 5a. OS - Overall cohort

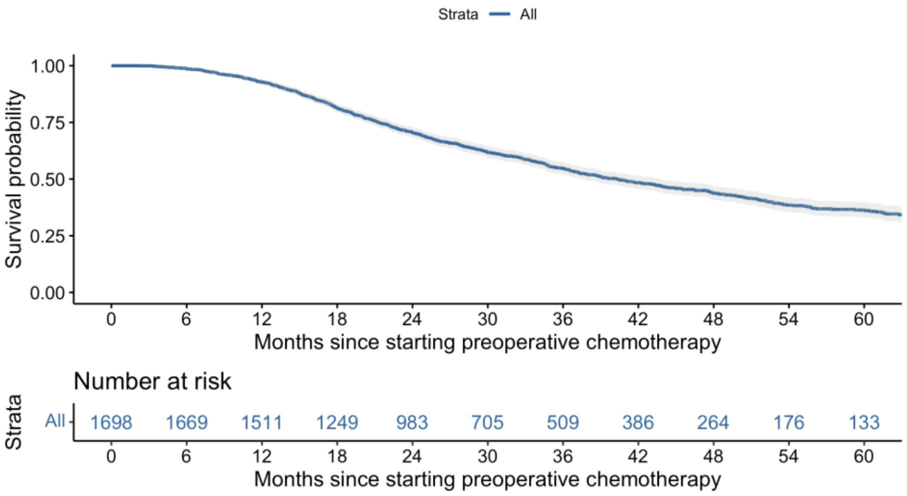

FIGURE 5b. OS - Patients with pCR *vs.* without pCR

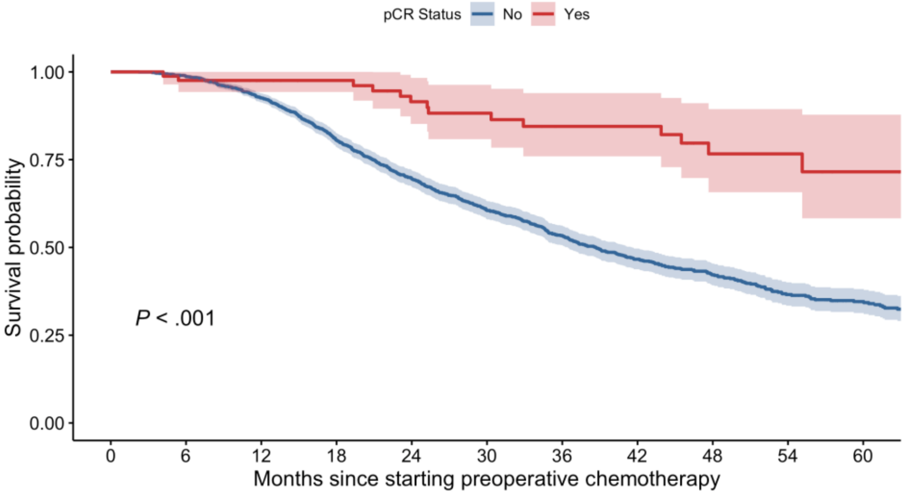

Overall survival (OS) measured from start of preoperative chemotherapy:
Figure 5a: mOS = 40 months (95%CI 37-44); 1-year OS = 93% (95%CI 92-94); 3-year OS = 55% (95%CI 52-58); 5-year OS = 36% (95%CI 33-40).
Figure 5b: Patients without pCR: mOS = 39 months (95%CI 36-42); 1-year OS = 93% (95%CI 91-94); 3-year OS = 53% (95%CI 51-56); 5-year OS = 34% (95%CI 31-38). Patients with pCR: mOS = not reached; 1-year OS = 98% (95%CI 94-100); 3-year OS = 85% (95%CI 76-94); 5-year OS = 72% (95%CI 58-88).

© 2024 Stoop TF et al. *JAMA Network Open.*

APPENDIX 6. Recurrence site in patients with disease recurrence

	Patients, No. (%)		
**Variables**	**pCR** ***n* = 21**	**No pCR** ***n* = 1015**	***P* value**
Locoregional recurrence	8 (38.1)	377 (37.1)	.10[c]
Distant recurrence	12 (57.1)	409 (40.3)	
Locoregional + distant recurrence	1 (4.8)	229 (22.6)	

pCR, pathological complete response; c, Fisher's Exact Test.

© 2024 Stoop TF et al. *JAMA Network Open.*

APPENDIX 7. Region-specific data

Variables	Patients, No. (%) Europe *n* = 594	United States *n* = 798	Japan *n* = 366
**Baseline characteristics**			
Age (mean ± SD) (years)	63 (10)	64 (9)	66 (9)
Resectability			
Primary resectable	171 (28.8)	164 (20.8)	94 (25.7)
Borderline resectable	314 (52.9)	397 (50.3)	145 (39.6)
Locally advanced	109 (18.4)	229 (29.0)	127 (34.7)
Missing	0	8	0
Tumor location			
Pancreatic head	423 (71.2)	592 (74.3)	261 (71.3)
Pancreatic body/tail	171 (28.8)	205 (25.7)	105 (28.7)
Missing	0	1	0
CA-19.9 (median, IQR)	181 (40-549)	192 (55-690)	167 (37-500)
Missing	200	99	25
CEA (median, IQR)	2.6 (2.2-4.2)	3.7 (2.2-6.2)	3.4 (2.1-5.7)
Missing	315	535	37
**Preoperative therapy**			
Chemotherapy*			
(m)FOLFIRINOX	315 (53.0)	459 (57.5)	23 (6.3)
Gemcitabine-nab-paclitaxel	110 (18.5)	229 (28.7)	162 (44.3)
Gemcitabine-S1	1 (0.2)	0 (0)	99 (27.0)
Gemcitabine-oxaliplatin	96 (16.2)	5 (0.6)	0 (0)
Other multi-agent regimens	26 (4.4)	60 (7.5)	4 (1.1)
Gemcitabine	46 (7.7)	27 (3.4)	24 (6.6)
S1	0 (0)	0 (0)	53 (14.5)
Other single-agent regimens	0 (0)	18 (2.3)	1 (0.3)
Second-line chemotherapy	21 (3.5)	142 (17.8)	53 (14.5)
Missing	0 (0)	0 (0)	74 (20.2)
Chemotherapy dose reduction	77 (13.0)	117 (14.7)	56 (15.3)
Missing	95 (16.0)	412 (51.6)	153 (41.8)
Radiotherapy			
No	377 (63.5)	233 (29.3)	261 (73.5)
Yes, conventional radiotherapy	206 (34.7)	168 (21.2)	88 (24.8)
Yes, SBRT	11 (1.9)	393 (49.5)	6 (1.7)
Missing	0	4	11
Preoperative therapy duration			
< 4 months	144 (24.2)	156 (19.5)	175 (47.8)
≥ 4 to < 6 months	165 (27.8)	224 (28.1)	80 (21.9)
≥ 6 to < 12 months	245 (41.2)	311 (39.0)	81 (22.1)
≥ 12 months	32 (5.4)	57 (7.1)	29 (7.9)
Missing	8 (1.3)	50 (6.3)	1 (0.3)
**Disease response**			
RECIST			
Complete response	8 (1.4)	5 (0.6)	1 (0.3)
Partial response	230 (39.0)	147 (18.6)	129 (35.9)
Stable disease	348 (59.0)	630 (79.7)	216 (60.2)
Progressive disease	4 (0.7)	8 (1.0)	13 (3.6)

© 2024 Stoop TF et al. *JAMA Network Open.*

Missing	4	8	7
CA-19.9 (median, IQR) (U/ml)	30 (12-71)	42 (18-115)	28 (11-72)
Missing	150	94	1
CEA (median, IQR) (U/ml)	2.5 (2.0-3.6)	3.1 (1.9-5.5)	3.3 (2.1-5.1)
Missing	259	545	1
**Histopathology**			
pCR	26 (4.4)	53 (6.6)	6 (1.6)
**Follow-up**			
Adjuvant chemotherapy	375 (63.1)	483 (60.5)	292 (79.8)
Missing	15 (2.5)	137 (17.2)	5 (1.4)
Median OS from surgery (months) (95%CI)	32 (30-39)	30 (27-37)	39 (33-46)
Missing	0	1	0
Median OS from start of preoperative chemotherapy (months) (95%CI)	41 (36-50)	37 (34-42)	46 (39-52)
Missing	8	51	1

SD, standard deviation; *ASA-PS,* American Society of Anesthesiologists Performance Status; *mm,* millimeters; *CA-19.9,* carbohydrate 19.9; *U,* units; *ml,* milliliters; *CEA,* carcinoembryonic antigen; *ng,* nanogram; *SBRT,* stereotactic body radiotherapy; *RECIST,* response evaluation criteria in solid tumors; *pCR,* pathological complete response; *OS,* overall survival.

Categorical data with missing data for ≥2% of patients were included in a separate category and were therefore included in the overall proportions and in the test of association. Otherwise, for missing <2%, the data are shown but were not included in the hypothesis tests.

NB. In some patients with pCR, a tumor size >0mm was registered. Because no vital tumor cells were detected, this suggests that the assumed 'former non-vital tumor tissue' is included in calculating the tumor size by the local pathologist.

NB. In some patients with pCR, data about lymphovascular invasion, perineural invasion, tumor differentiation, and/or residual disease is missing. This data is handled as missing, although the absence of lymphovascular and perineural invasion, impossibility to determine differentiation, and no residual disease in the resection margins can be assumed.

© 2024 Stoop TF et al. *JAMA Network Open.*
