# Supplementary material for: Pathological Complete Response in Patients With Resected Pancreatic Adenocarcinoma After Preoperative Chemotherapy
Source: JAMA Netw Open. 2024 Jun 18;7(6):e2417625. doi: 10.1001/jamanetworkopen.2024.17625 (PMC11185983; doi:10.1001/jamanetworkopen.2024.17625)
Supplement: Supplement 2. — Data Sharing Statement [file jamanetwopen-e2417625-s002.pdf]

# Data Sharing Statement

Stoop. Pathological Complete Response in Patients With Resected Pancreatic Adenocarcinoma After Preoperative Chemotherapy. *JAMA Netw Open*. Published June 18, 2024. doi:10.1001/jamanetworkopen.2024.17625

**Data**
**Data available:** No

**Additional Information**
**Explanation for why data not available:** In this multicenter collaboration, de-identified data from each participating centers is confidentially shared with the leading institution (University of Colorado).
